# A "Hut" Hospital

**Published:** 1917-06-09

**Authors:** Ward Muir

**Affiliations:** R.A.M.C.T., 3rd London General Hospital


### A "HUT" HOSPITAL.

By LANCE-CORPORAL WARD MUIR, R.A.M.C.T., 3rd London General Hospital.

### How the 3rd London General Hospital came into being.

People have curious ideas of the kind of building which would make a good war hospital. "The So-and-So Club in Pall Mall," I have been told, "should have been commandeered long ago. Ideal for hospital purposes. Of course, some of the M.P. members brought influence to bear, and the War Office was choked off. . . ." and so forth.

It would surprise me to hear of anything that the War Office was held back from doing if it wanted to do it. Perhaps the least likely obstructionist to be successful in this project would be a club-frequenting M.P. The War Office has taken exactly and precisely what it chose—even when it would have been better to choose otherwise. In this matter of commandeering buildings for hospitals it may or may not have acted with wisdom; but at least it has been safe in avoiding the advice of the individual who jumps to the conclusion that just any pleasingly situated edifice will do, provided beds and nurses are shovelled into it in sufficient quantities.

The indignant patriot who was convinced that chicane alone saved the So-and-So Club from being dedicated to the service of the wounded was quite unable to tell me whether the lifts—assuming that lifts existed—were roomy enough to accommodate stretchers; whether, if so, no interval of stairs prevented trollies from being wheeled to every ward; whether the arrangement of the building would allow of the network of plumbing necessitated by the introduction of numerous bathrooms and lavatories (for each ward must possess both); whether the kitchens were so located that they could supply food to top-floor patients without waste of carrying labour on the part of the orderlies' staff. These problems, the mere fringe of the subject, had never occurred to our patriot. His idea of a hospital was a place where soldiers lie in bed and get well. (What queer notions visitors absorb of the *easiness* of hospital life!) He had not glimpsed the organisation which made the cure possible. The man in bed, a sister hovering in the background with apparently nothing to do but look pleasant—these constituted for him the final phenomena of a war hospital. These phenomena, instead of being housed in a wood and corrugated-iron shed, might have been staged picturesquely in one of the luxurious salons of the So-and-So Club in Pall Mall. It was a shame that they weren't. He would write to the papers about it. Somebody must be blamed, somebody must be made to hustle. And meanwhile the sisters and doctors who *were* installed in gorgeous mansions for their work were openly envying the fortunate ones who had been given those bare but efficient and compactly planned sheds.

Some years ago a number of public buildings were earmarked for hospital use in case of war. It may surprise the indignant patriots to learn that any preparations whatever were made prior to the outbreak in 1914. Nevertheless all kinds of preparations actually were made. Mistakes and miscalculations may have marred those preparations; the fact remains that, as far as the Territorial Medical Service was concerned, the authorities had merely to press a button and hospitals came into existence. Thus a number of institutions—mostly schools—found themselves ejected from their own rooftrees: found, in short (what many other folk were to learn later), that the State is omnipotent in wartime and that sectional interests fade into insignificance compared with the interests of the safety of the commonwealth. Some conception of the promptness with which this paper scheme of Sir Alfred Keogh's materialised at the outbreak of war may be gathered from the simple statement that the building of which I myself write was an Orphans' Home on August 4, 1914. At 6 A.M. on August 5 it was a military hospital.

I do not say that it was a military hospital in working order. But if, by a miracle, wounded *had* turned up then, there was at least a staff of medical officers and orderlies on the premises to receive them. In point of fact it was some weeks before the first patients arrived. Those weeks, however, were not idle ones. The layman who considers that any large building can be turned instantaneously into a hospital would have had an eye-opener if he had witnessed the work done here. The mere removing of 95 per cent. of the institution's furniture was a colossal task: added thereto was the introduction of hundreds of beds, hundreds of mattresses, hundreds of sets of bedclothes, hundreds of suits of pyjamas, hundreds of—— But why prolong a brain-racking list? Then there was the pulling down and fixing up of partitions, the removal of every single window for replacement by hopper sashes, the fitting-in of bathrooms, lavatories, ward-kitchens, sink-rooms, dispensary, cook-house, operating theatre, pathological laboratory, linen store, steward's store, clothing store, detention-room, administration offices, *x*-ray department . . . all these in a building which, spacious and handsome outwardly, was, as to its interior, a characteristic maze in the Scottish baronial style of architecture beloved by mid-Victorian philanthropists. How the evicted orphans will like to return to those stone-flagged passages and large airy dormitories after having experienced the comforts of the banal but snug suburban villas in which they are at present located, I know not. There is a certain dignity about the Scottish baronial pile, I admit. The silhouette of its grey stone façade, rising above delightful lawns, makes a good impression—from a distance. Postcard views of it sell freely to visitors. But the best part of our hospital is hidden behind that turreted façade, and is much too "ugly" and utilitarian for postcard immortalisation.

The best part of our hospital—*the* hospital, to most of us—came into being when the commandeered Scottish baronial orphans' asylum was found to be too small. Then were built "the huts." The word "hut" suggests something casual, of the camping-out order: a shed knocked together with tin-tacks, doubtfully weatherproof, and probably scamped by profiteering contractors. Of the huts provided at certain training centres this may have been true. The finely austere and efficient ranks of hut-wards which constitute the main part of the 3rd London General Hospital are the very antithesis of that picture. They may look flimsy. They were certainly put up at a remarkable pace. I myself witnessed the erection of the final fifty of them. An open field vanished in less than a month, and "Bungalow Town" (as someone nicknamed it) appeared. You would have said that such speed meant countless imperfections of detail. No doubt some tinkerings and modifications were bound to follow, when the regiment of workmen, carpenters, engineers, drainage specialists, electricians, had vanished. But, in the long run, the ideal hospital remained—a hospital with which the So-and-So Club in Pall Mall, for all its luxuriousness, could never hope to compare.

There are still a dozen wards—used mostly for medical cases—in the Scottish baronial building. Its rooms, too, provide the administration with offices. Its great dining-hall is a splendid receiving ward for the sorting out and clearance of newly-arrived convoys of patients. We should be poorly situated indeed if we had not our Scottish baronial main building to be the hub of the hospital's activities, or rather the handle from which springs the fan of the hospital's great extension—the huts. Approaching the hospital, the visitor sees nothing of those huts. As he walks up the drive he flatters himself that he has reached his destination. He discovers his mistake when, at the inquiry bureau in the entrance, he is informed that the patient whom he has come to interview is (say) in "C 13". He is advised to go down the passage on his left, turn to his right, turn to the left again, and then again to the right—after which he had better seek a further re-direction. Launching himself optimistically on this voyage he learns, long ere he has attained his goal, that a modern war-hospital can hide a considerable extent of pedestrianism behind a comparatively short Scottish baronial frontage. He will be fortunate if five minutes' steady tramping brings him to the bedside of his friend in C 13.

Perhaps he will comfort himself in his footsoreness by noting that, to reach C 13, he has not had to go up or down any stairs. This is one of the beauties of the hut system. It consumes a big area, but it is all on one level—the ground level. The patient on crutches can go anywhere without fear of tripping, the patient in a wheeled chair can propel himself anywhere, the orderlies can push wheeled stretchers or dinner-wagons anywhere. Our visitor for C 13 having escaped from the back of the Scottish baronial building, emerges into a vista of covered corridors, wooden-floored, galvanised-iron roofed. It is a vista which seems to have no end. Corridor branches out of corridor—A corridor, B corridor, C corridor, D corridor, each with its perspective of doors opening into wards; and shorter corridors leading to store-rooms and the like. But the patient or orderly who has dwelt in a hospital where, though distances are shorter, staircases are involved—or where every trifling coming and going of goods or stretchers necessitates the manipulation of a lift—blesses those level, smooth corridors, with their facile access to any ward, to operating theatres, kitchens, stores, *x*-ray room, massage department, etc., and their stepless exit into the open air.

Looked at from outside, a hut-ward is—to the æsthetic eye—a hideous structure. Knowing what it stands for, the science, the tenderness, and the fundamental civilisation which it represents, we may descry, behind its stark geometrical outlines, a real nobility and beauty. Entering a typical hut ward you behold thirty beds, fifteen on each side of the room. Between each pair of beds is a locker in which the patient stows his belongings. In the central aisle of the room are the sister's writing-table, certain other tables, chairs, and two coke stoves for heating purposes in winter. The floor is carpetless, and maintained in a meticulous state of high gloss by means of daily polishings. At a height of a few feet from the floor, the asbestos-lined walls cease, and become windows. There is no gap in the continuous line of windows all down each side of the ward—a special type of window which, even when open, declines to allow rain to enter. In consequence of these windows the ward is not only very well lit, but also airy and odourless. When all the windows are open (which is the case throughout the entire summer and generally the case in winter also), the patient has the advantage of indoor comfort plus an outdoor atmosphere. At the end of the ward a covered verandah is spacious enough to take an extra couple of beds for those requiring completely open-air treatment.

The ward proper has certain additions; a kitchen with gas-stove and geyser, a sink-room with geyser and cleansing apparatus of special pattern, a bathroom with geyser, lavatories, a small room for the isolation of a patient on the danger list, a linen-room, and cupboards. All these are packed neatly under that one rectangular corrugated roof which looked so ugly and so unpromising from outside.

Do not pity the wounded soldier because he is quartered in a "hut." The word sounds unattractive. But if it is the right kind of hut, he is in the soundest and most sanitary type of temporary hospital that the mind of man has yet devised. The raindrops may rattle a shade noisily on the roof, the asbestos lining may be devoid of ornamentation, but as he lies in bed and contemplates that unadorned ceiling he is a deal better off than if he were gazing at the elaborate (and dust harbouring) cornices of the So-and-So Club's grandiose smoking lounge in Pall Mall.

